# Evergreen Needle Magnetization as a Proxy for Particulate Matter Pollution in Urban Environments

**DOI:** 10.1029/2020GH000286

**Published:** 2020-09-10

**Authors:** Grant Rea‐Downing, Brendon J. Quirk, Courtney L. Wagner, Peter C. Lippert

**Affiliations:** ^1^ Department of Geology and Geophysics University of Utah Salt Lake City UT USA; ^2^ Now at the Department of Geosciences University of Massachusetts Amherst MA USA; ^3^ Global Change and Sustainability Center University of Utah Salt Lake City UT USA

**Keywords:** particulate matter, evergreen needles, environmental magnetism, air pollution, proxy, biomagnetic monitoring

## Abstract

We test the use of magnetic measurements of evergreen needles as a proxy for particulate matter pollution in Salt Lake City, Utah. Measurements of saturation isothermal remanent magnetization indicate needle magnetization increases with increased air pollution. Needle magnetization shows a high degree of spatial variability with the largest increases in magnetization near roadways. Results from our magnetic measurements are corroborated by scanning electron microscopy of needle surfaces and by inductively coupled plasma mass spectrometry of metal concentrations in residues collected from sampled needles. Low‐temperature magnetic analysis suggests the presence of small (<20 nm) partially oxidized magnetite particles on needles collected adjacent to a major roadway. Magnetization may be a low‐cost proxy for certain metal concentrations (including lead) during periods of increased particulate pollution. The spatial resolution of our method appears capable of resolving changes in ambient particulate matter pollution on the scale of tens to hundreds of meters. Questions remain regarding the timescales over which evergreen needles retain particulate matter accumulated during atmospheric inversion events in Salt Lake City. Results presented here corroborate previous studies that found needle magnetization is a fast, cost‐effective measure of particulate matter pollution. This method has the potential to provide high spatial resolution maps of biomagnetically monitored particulate matter in polluted urban environments year‐round.

## Key Points

- Needle magnetization is highest proximal to major roadways and varies on a spatial scale of tens to hundreds of meters
- Changes in magnetization correlate with changes in concentration of several toxic metals measured from the surfaces of needles
- Evergreen needles are capable of recording severe periods of degraded air quality that last days to weeks

## 1 Introduction

Air pollution containing particulate matter (PM) is a major public health concern in large population centers around the world (Dai et al., 2014; Kheirbek et al., 2016; Lin et al., 2016). Fine particulate matter is linked to a wide range of health concerns, including respiratory, cardiovascular, and neurodegenerative diseases (Atkinson et al., 2001; Maher, 2019; Pope et al., 2015). Some urban regions are affected by air pollution associated with seasonal atmospheric temperature inversions. Atmospheric inversions occur when warm air sits above cooler, dense air and inhibits typical atmospheric circulation (Whiteman et al., 2004). As a result of these atmospheric temperature inversions, pollutants concentrate near the ground surface and can lead to multiday periods of severely degraded air quality, referred to as poor air quality events (Olofson et al., 2009). When atmospheric inversion occurs over urban environments, large populations become at risk for myriad health complications tied to poor air quality (Trinh et al., 2019).

Salt Lake City, UT, experiences enhanced winter‐time air pollution which is attributed to fossil fuel combustion and industry combined with atmospheric inversions (Baasandorj et al., 2018). These inversion events cause particulate matter smaller than 2.5 μm in diameter (PM_2.5_) to accumulate in high concentrations in the air. Multiple studies document the severe respiratory, neurological, and perhaps cognitive health risks associated with exposure to high concentrations of PM_2.5_ (Lakey et al., 2016; Maher, 2019; Underwood, 2017). These health risks increase as the concentration of the finest metal‐bearing particles within the PM_2.5_ fraction increases (e.g., Maher et al., 2016). Therefore, there is a critical need to understand the temporal and spatial distribution of PM throughout an affected population center. Characterizing the distribution of particle sizes within metal‐bearing PM—especially within the PM_2.5_ size range—is a key component of mapping PM in urban environments. Most city centers have only a few PM monitoring stations active at any given time, and the spatial resolution of airborne PM is correspondingly poor. This shortage of monitoring persists despite several studies that emphasize the high degree of spatial variability in air quality and PM in urban environments (Krasnov et al., 2016; Mendoza et al., 2019; Thai et al., 2008; Wilson et al., 2005).

The successful use of plants as biomonitors of polluted environments has been demonstrated in multiple studies (e.g., Bargagli, 1998; Markert et al., 2003). Environmental magnetic analysis of biological samples (i.e., biomagnetic monitoring) has been thoroughly documented as a robust indicator of atmospheric particulate pollution in a variety of settings (Jordanova et al., 2003, 2010; Lehndorff et al., 2006; Muxworthy et al., 2003; Urbat et al., 2004). Several studies show how the magnetization of deciduous tree leaves is a low‐cost, high‐resolution approach for determining the spatial distribution of PM in urban environments (Hanesch et al., 2003; Hofman et al., 2013, 2017; Maher et al., 2008). However, the use of deciduous tree leaves for monitoring PM is seasonally limited: intermontane urban areas such as Salt Lake City are especially susceptible to winter‐time poor air quality events. The use of evergreen needles as biomonitors of particulate matter pollution has shown promise in previous work. For example, Urbat et al. (2004) and Lehndorff et al. (2006) showed how magnetic susceptibility and remanence properties of *Pinus nigra* needles vary at different sampling locations in accordance with proximity to pollution sources across a broad urban area (Cologne, Germany). Similarly, Lehndorff and Schwark (2008, 2010) documented the major and trace element concentrations of PM on and within the needles. Here we present results of magnetic, chemical, and electron microscopic analysis of evergreen needles sampled on the University of Utah campus during an inversion event in Salt Lake City, UT. We compare inversion samples to samples taken from the same locations during optimal air quality conditions. This approach allows us to assess the effectiveness of the magnetization of evergreen needles as a year‐round, cost‐effective measure of metal‐bearing PM in air pollution in Salt Lake City.

## 2 Methods

### 2.1 Sampling

**Figure 1:**
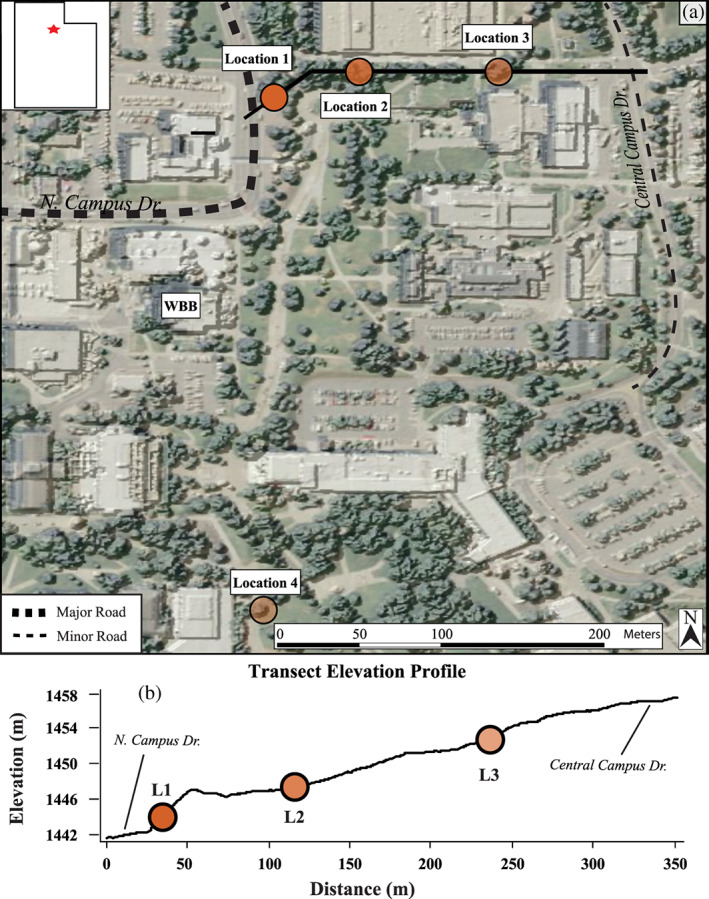
(a) Map of the University of Utah campus showing sample locations (orange circles) and elevations profile transect (black line) as well as major (>13,000 annual average daily traffic, AADT) and minor (<10,000 AADT) campus roads. WBB = William Browning Building. (b) Elevation profile of sample transect with Locations 1–3 indicated.

We collected approximately 50 needles per sample from Austrian pines (*P*. *nigra*) growing on the University of Utah campus (Figure 1). We collected samples on 14 June 2017 following several months of good air quality. We sampled again on 15 December 2017 during peak atmospheric temperature inversion and a corresponding poor air quality event. We chose each sampling location to test the relationship between road proximity and PM concentration. Location 1 is immediately adjacent to North Campus Drive, a major campus and city roadway (~13,000 Annual Average Daily Traffic; Utah Department of Transportation, 2017). Location 2 is approximately 50 m east of Location 1, and Location 3 is approximately 100 m east of Location 2 near a second, less traveled roadway (Figure 1). Location 4 is a control site away from major roadways and in a region of campus with the least motorized vehicle traffic. We collected samples at a height of approximately 1.5 m from the ground surface and from the southern side of each tree midway between road‐proximal and road‐distal positions in the case of the road transect (Locations 1–3). Samples were preferentially taken from needles older than the 2017 needle cohort although no attempt was made to distinguish between older cohorts. *P*. *nigra* retains needles for between 1 and 3 years.

**Figure 2:**
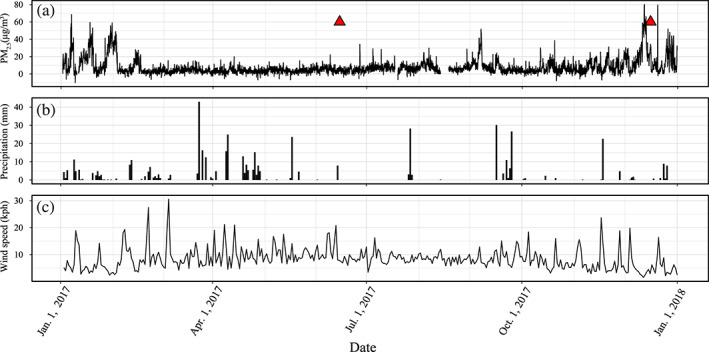
(a) PM_2.5_ concentrations measured for the Salt Lake valley for the year of 2017. The monitoring site (40.7343°N, 248.1278°W) from which data were collected is ~5 km southwest and 100 m lower than the sampling locations shown in Figure 1. Red triangles indicate sampling periods in this study. (b, c) Precipitation and wind speed data for the year of 2017 as measured at a weather station on the roof of WBB (Figure 1, data available: https://mesowest.utah.edu/) ~100 m south and 40 m higher than sample Location 1.

Figure 2 shows PM_2.5_ concentrations, precipitation, and wind speed data in Salt Lake City for the entire year; our two sampling periods are indicated in this time series. PM_2.5_ levels averaged ~10 μg/m^3^ in the 3.5 months prior to our June sampling, and there were no major pollution events during this interval. In contrast, PM_2.5_ levels spiked immediately prior to our December sampling: PM_2.5_ reached the highest levels of 2017 (>60 μg/m^3^) during this event, and concentrations remained high for most of the week prior to December 15. The PM_2.5_ data displayed in Figure 2a come from the Hawthorne Elementary school monitoring site, which is approximately 5 km to the southwest and 100 m lower elevation than our sample locations. This site is used by state and local agencies in the Salt Lake valley to determine if local air quality meets national standards. The precipitation and wind speed data in Figures 2b and 2c were obtained from a weather monitoring station on the roof of the William Browning Building (WBB, Figure 1) approximately 100 m south and 40 m above Location 1. These data show that there were no precipitation or wind events between peak poor air quality conditions and sampling in December; there was a large precipitation event (~25 mm) on 17 November 2017, 20 days prior to the onset of poor air quality conditions. Wind and precipitation were highest in the months preceding June sampling with average wind speed of ~10 kph from March through June and frequent, >10 mm precipitation events over the same period.

### 2.2 Magnetic Measurements

All room temperature magnetic measurements were completed at the Utah Paleomagnetic Center at the University of Utah. Individual needles were cut into ~10 mm lengths using nonmagnetic ceramic scissors and packed into 10 cc plastic sample cubes; each sample was handled with new, clean gloves and the scissors washed between each sample preparation. Each sample was subjected to stepwise isothermal remanent magnetization (IRM) using an ASC Scientific IM‐10‐30 Impulse Magnetizer. Fields of 0, 20, 50, 100, 300, 650, and 1,000 mT were applied to each sample. The resulting magnetization of each sample was measured on a 2G Enterprises Model 760‐3 DC SQUID rock magnetometer after each step and normalized by sample mass.

Low‐temperature magnetic measurements were completed at the Institute for Rock Magnetism at the University of Minnesota, Twin Cities, using two Quantum Designs Magnetic Property Measurement Systems. The low‐temperature measurement routines described below follow Bilardello and Jackson (2013) and their protocols for field‐cooled (FC), zero‐field‐cooled (ZFC) low‐temperature (LT) saturation of isothermal remanence magnetization (SIRM), and low‐temperature cycling of room‐temperature SIRM (RT‐SIRM) experiments. Samples were cooled from room temperature to 20 K in the presence of a 2.5 T magnetic field after which their remanence was measured every 5 K as the sample warmed to room temperature (FC‐LT‐SIRM). Samples were then cooled in the absence of an external field to 20 K at which point a 2.5 T field was again applied to the samples after which their remanence was again measured as they warmed to room temperature (ZFC‐LT‐SIRM). Finally, a 2.5 T field was applied to the samples at room temperature, and their remanence was measured as they cooled to 20 K and then warmed to room temperature (RT‐SIRM). We completed these low‐temperature magnetic remanence measurements only on noninversion and inversion samples from Location 1.

### 2.3 Elemental Analysis

Elemental analysis was completed at the University of Utah ICP‐MS Metals Laboratory. Needles from all sample locations and events were leached in a 2.4% HNO_3_ solution for 72 hr following procedures described in Maher et al. (2008). The leachate was measured on an Agilent 7500ce quadrupole mass spectrometer. We measured 42 individual metal concentrations ranging from alkali metals to metalloids (see Table S1 in the supporting information for a complete list). In order to assess the efficacy of acid leaching, a second aliquot of inversion‐event needles from Locations 1 and 4 were “cleaned” via sonication in “MilliQ” water for 5 min to compare against the acid leach from the previous aliquot. We also measured the metal concentrations within microwave digested needles that had been previously acid leached; these analyses were completed only for samples from Locations 1 and 4. In summary, we measured a total of six aliquots from Location 1: two aliquots from acid leaching (inversion and noninversion), two aliquots from sonication (inversion and noninversion), and two aliquots from microwave digestion of previously acid leached samples (inversion and noninversion). The same routine was followed for Location 4. From Location 2, we measured two aliquots: acid leach solutions from both inversion and noninversion samples. The same routine was followed for Location 3.

### 2.4 Electron Microscopy

Scanning electron micrographs were obtained at the Surface Analysis Lab at the University of Utah. Samples were prepared by cutting ~1 cm long segments from the middle of four to six needles from each sampling location for both noninversion and inversion samples. These segments were coated with ~60 nm of carbon and grounded to a standard SEM stub with copper tape. Images were collected on a FEI Quanta 600F SEM under high and low vacuum with an accelerating voltage of 10–20 kV and a working distance of ~10 mm. We obtained approximately 90 images of needles collected at each of the four sampling locations for both noninversion and inversion samples.

## 3 Results

### 3.1 IRM Acquisition

**Figure 3:**
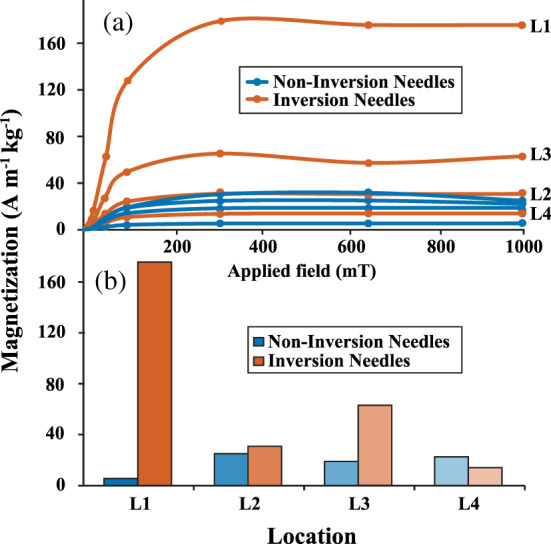
(a) Isothermal remanent magnetization acquisition curves for inversion (orange) and noninversion (blue) needles. Measurements were made after applying 0, 20, 50, 100, 300, 650, and 1,000 mT fields to each sample. (b) Saturation of isothermal remanent magnetization values for each sample after application of a 1,000 mT field.

Both noninversion and inversion evergreen needles show large increases in magnetization in fields below 100 mT and reach isothermal remanent magnetization saturation (SIRM) at ~300 mT (Figure 3a). Three of the four inversion samples (Locations 1, 2, and 3) acquired a stronger SIRM than its corresponding noninversion sample. This difference was largest in samples from Locations 1 and 3, for which magnetization increased by factors of ~30 and 3, respectively (Figure 3b). Location 2 also acquired a larger SIRM value during inversion, but this increase was moderate (factor of ~0.25). In contrast to the other samples, the SIRM values for Location 4 decreased during inversion. SIRM changes at both Locations 2 and 4 are small in comparison to Locations 1 and 3, and we note that these changes are small enough that they could be the result of variability in the needles collected at those sites or even measurement variability. Noninversion samples yielded lowest SIRM values at Locations 1 and 3. None of the noninversion samples achieved magnetizations greater than 25 A m^−1^ kg^−1^; this is almost an order of magnitude lower than the maximum inversion SIRM at Location 1 (175 A m^−1^ kg^−1^).

### 3.2 Low‐Temperature Remanence

**Figure 4:**
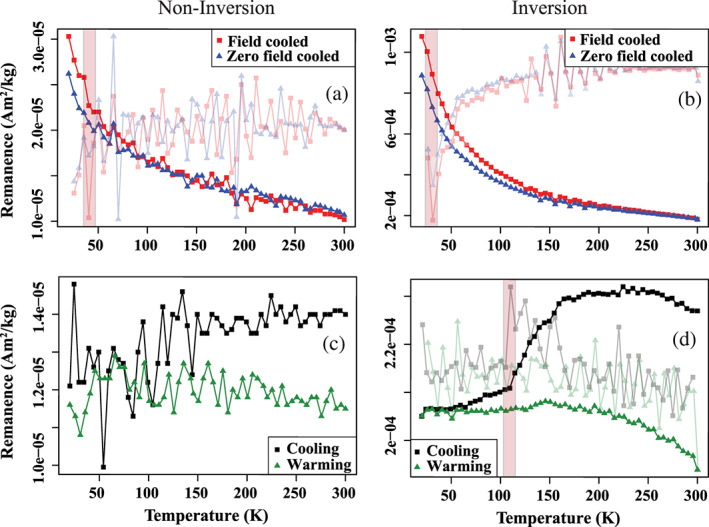
Field cooled (FC) and zero field cooled (ZFC) low‐temperature saturation of isothermal remanence magnetization (LT‐SIRM) curves for Location 1 sampled noninversion (a) and during inversion (b): FC‐ZFC‐LT‐SIRM. (c, d) Low‐temperature cycling of a room temperature saturation isothermal remanent magnetization for Location 1 sampled noninversion (c) and during inversion (d): RT‐SIRM. Derivative of each curve is shown in the background of each plot; a detailed description of each procedure is provided in Section 2.2.

Low‐temperature SIRM and low‐temperature cycling of room temperature SIRM measurements for noninversion and inversion samples collected at Location 1 are shown in Figure 4. The low‐temperature magnetization of the inversion sample is an order of magnitude greater than that for the noninversion sample, similar to the results from the room temperature measurements: 2.3E−04 A m^2^ kg^−1^ versus 1.4E−05 A m^2^ kg^−1^. Field‐cooled (FC) and zero‐field‐cooled (ZFC) low‐temperature SIRM curves for both samples show a rapid, nonlinear decrease in remanence between 20 and ~150 K. FC remanence is higher than ZFC remanence at every temperature step in the inversion sample and at low temperatures in the noninversion sample; measurement noise is larger in the noninversion measurement. The derivatives for both FC and ZFC curves yield minima at ~30 K for the inversion sample. A similar minimum may be present in the noninversion sample, but the greater measurement noise makes such a conclusion uncertain. We also observe a dramatic decrease in measurement noise as the inversion sample cools below ~120 K; this is particularly clear in the derivative curves for FC and ZFC experiments through this interval.

Low‐temperature cycling of a room temperature SIRM yields a “hump” shaped remanence curve for the inversion sample between 300 and ~150 K. This distinctive behavior in the cooling curve is followed by a steady, steep decay of remanence until ~120 K at which point there is an abrupt inflection point below which remanence decays more gently. The “hump” shape is seen in the warming curve between 150 and 300 K, but the inflection point at 120 K is absent. The magnetization of the noninversion sample is much weaker, and the reduced signal makes interpretation of its low‐temperature remanence behavior difficult. Nevertheless, we observe a convergence of the cooling and warming curves below ~150 K.

### 3.3 Chemistry

**Figure 5:**
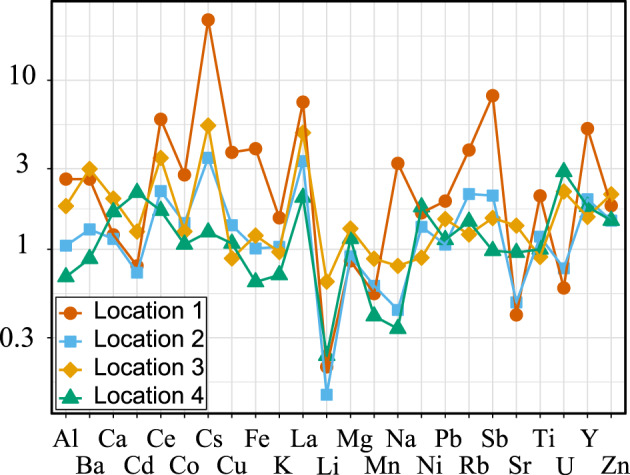
Enrichment factors as calculated in comparison to the noninversion sample from Location 4 for each location for metal concentrations that were above instrument detection limits. Concentrations plotted on logarithmic scale for increased clarity. Explanation for how enrichment factors were calculated are provided in Section 3.3.

Elemental concentrations for the 42 measured metals are listed in the supporting information for each of the acid leached samples described above (see Figure S1 and Table S1 in the supporting information). We calculate the relative enrichment of metals at each site by dividing each inversion event concentration by the noninversion concentration measured at Location 4 (the “background” site). This is in contrast to how enrichment factors are typically calculated with respect to soil or dust values. We calculated metal concentrations with respect to needle mass. The challenge with using a more traditional approach to calculating enrichment factors is that the surface area‐to‐mass ratio is far smaller for needles than dust or soil samples and all measured metal concentrations are several orders of magnitude smaller than soil (Waddell & Giddings, 2004) and dust (Goodman et al., 2019) samples measured in the Salt Lake region. Another approach would be to compare our concentrations directly to previously reported needle data. Although other studies have measured metal concentrations of PM deposited on needle surfaces, these values vary widely between species and location (Lehndorff & Schwark, 2010). Thus, we elected to compare our results to our local “background” sample, similar to the approach used by Maher et al. (2008). Concentrations increase for almost every measurable element during inversion at Locations 1 and 3 (Figure 5). Metal concentrations at Locations 2 and 4 are more variable and show both higher and lower concentrations of different elements during inversion. These two locations are characterized by a more muted response to the inversion event than observed at Locations 1 and 3 (Figure 5). Cs, Rb, Cu, Y, Ce, Ti, Co, Pb, La, Ba, Al, Fe, and Sb show the largest increase in concentration during inversion at Locations 1 and 3.

We tested for correlation between SIRM and elemental results for which we had results above detection limits at all locations using the Pearson product‐moment statistical test. We made these calculations for both inversion and noninversion samples (see Figure S1 and Table S2 for complete results). Sample size is low for each sample set (*n* = 4), and therefore, statistical results should be interpreted with care; nevertheless, our small data set shows a strong correlation between SIRM and iron concentration (*R* = 0.99, *p* = 0.01 for inversion samples, *R* = 0.97, *p* = 0.03 for noninversion samples). Inversion samples also show significant correlation between SIRM and Na, Al, Cs, La, Ce, Pb, Sb, and Co (*R* ≥ 0.9, *p* ≤ 0.05). Noninversion elemental results show fewer significant correlations with respect to SIRM: only Al, La, and Mg (*R* ≥ 0.9, *p* ≤ 0.05) are strongly correlated.

**Figure 6:**
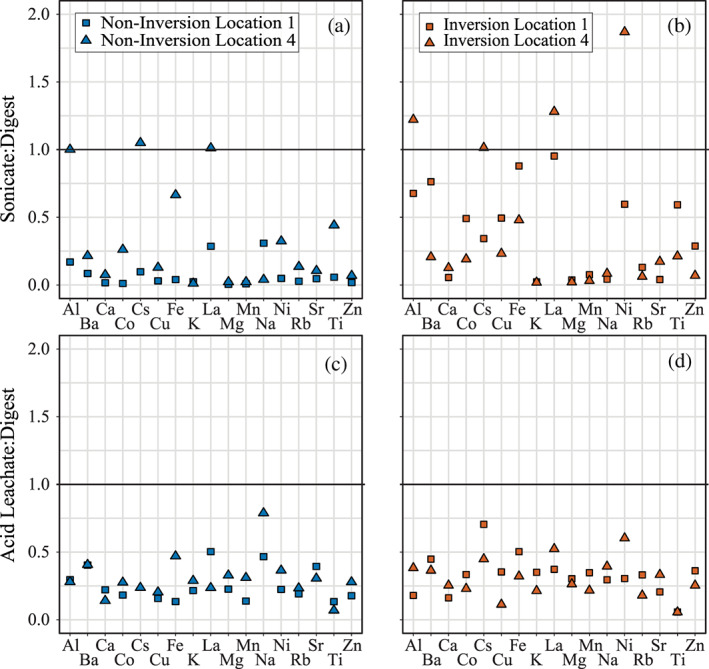
Comparison of the various methods devised to measure elemental concentrations of sampled needles. (a, b) Ratio comparing sonication of evergreen needles *vs.* total microwave digestion of previously acid leached needles collected noninversion (a) and during inversion (b) from Locations 1 and 4. (c, d) Ratio comparing acid leaching of evergreen needles *vs.* total microwave digestion of previously leached needles collected noninversion (a) and during inversion (b) from Locations 1 and 4.

Analytical results for sonication and total needle digestion for samples from Locations 1 and 4 are listed in Table S3. We compare these results with acid leach results at the same locations in Figure 6. Leachate and sonicate results are normalized by total needle digestion results in Figure 6 to directly compare each method at both locations during noninversion and inversion: values below 1 in Figure 6 indicate that sonication, acid leaching, or both fails to remove more than half of the metals present. The most obvious result is that neither “cleaning” technique (acid leach nor MilliQ Sonication) appears to remove the majority of the metals from the needles: average leachate:digest and sonicate:digest values are 0.43 and 0.30, respectively). On average, results from this study show that acid leaching is more effective at removing material from needles than sonication. That said, sonication does remove more of certain metals: more than 50% of material in the case of the four metals with sonicate:digest values >1 (Al, Cs, La, Ni). In general, our results show no appreciable differences between the cleaning techniques between inversion/noninversion samples or sample Locations 1 and 4. We note that these results hold only for analyses for which each method returned concentrations above instrument detection limits. Overall, the number of analyses below instrument detection limits was greatest for the acid leaching (52 of 168 analyses, Locations 1 and 4) and least for needle digestion (nine of 168 analyses, Locations 1 and 4).

### 3.4 Electron Microscopy

**Figure 7:**
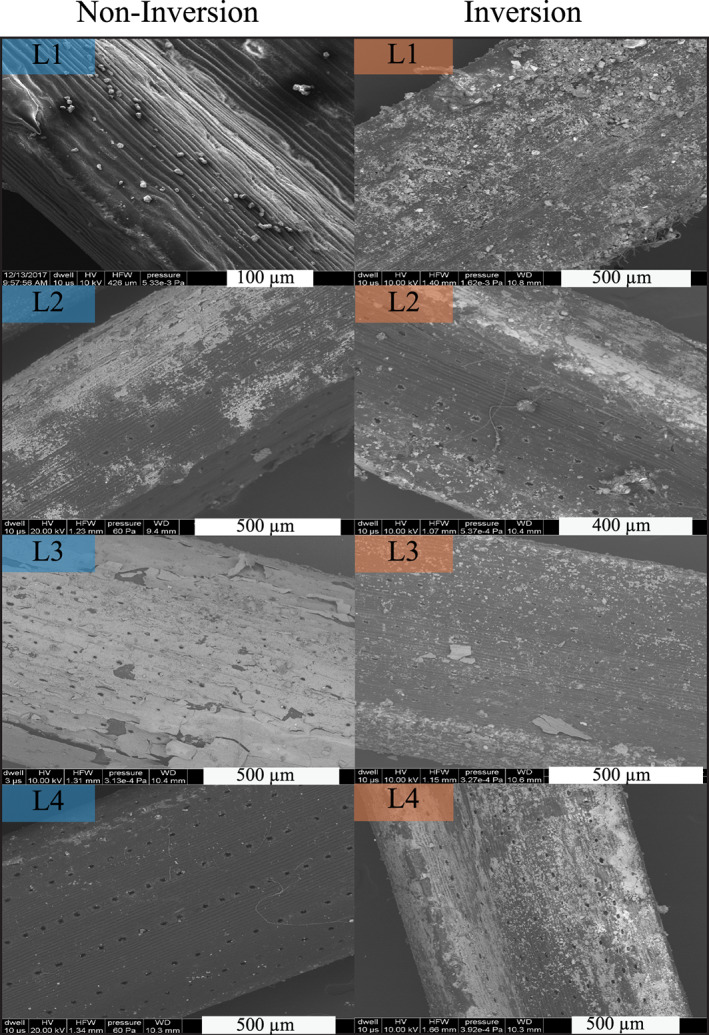
Scanning electron photomicrographs of evergreen needle surfaces. Images from each location are shown both during noninversion (left) and inversion (right).

Scanning electron micrographs of needles at each sample location during noninversion and inversion are shown in Figure 7. Images of the noninversion needles show little particulate matter on needle surfaces at each location at this scale of observation: stomata are clearly visible on needles from all locations, and only Location 1 shows even minor particulate matter on needle surfaces. By contrast, images of inversion needles show needle surfaces coated in fine particulate matter ranging from greater than 100 μm to less than ~10 μm. Location 1 is the most contaminated with particulate matter. Stomata are easily recognized and visible on needles from Locations 2 and 4, but they are almost entirely obscured by particulate matter at Location 1.

## 4 Discussion

### 4.1 Needles as Passive Air Quality Samplers

Previous work suggested that needle retention of particulate matter is governed by several environmental factors including wax content, stomatal uptake, precipitation, wind abrasion, and degree of particulate matter already present (Chen et al., 2017; Horstmann & McLachlan, 1998; Przybysz et al., 2019; Urbat et al., 2004). Urbat et al. (2004) found no correlation between epicuticular wax content and magnetic susceptibility of *P*. *nigra* needles collected from Cologne, Germany. They did, however, document a decrease in needle wax content in the warm summer months and a decrease in the magnetic susceptibility of needles in the late summer (August). We did not measure wax content directly as part of our study, but we note that the SIRM values of the needles collected in the summer are much lower than those collected in the winter. One possible explanation for this is lower wax content and thus lower PM retention during the summer. Precipitation and wind abrasion are both thought to reduce the amount of PM retained on needle surfaces via stripping and washing of surface‐coating PM. Average wind speed and precipitation were highest in the months leading up to our June sampling (Figures 2b and 2c), and these weather events could also contribute to the overall lower SIRM and metal concentrations measured on those samples. In contrast, there is a consistent decrease in wind speed and an absence of any precipitation in the lead up to the inversion and poor air quality event we sampled on 15 December 2017. This absence of PM‐stripping processes could increase needle retention of PM and may have contributed to the higher SIRM and metal concentrations observed for those samples. While these processes undoubtedly contribute to some seasonal variability in PM retention on evergreen needles, our results indicate that evergreen needles can serve as passive air quality sensors during poor air quality events when particulate matter pollution loading is especially high. The dramatic increase in the magnetization of needles and the scanning electron micrographs that clearly show particulate‐laden needles at Locations 1 and 3 document that iron‐bearing particulate matter is deposited and retained on the surfaces of pine needles during inversion events.

The correlation between SIRM values and proximity to major roadways on campus suggests that automobile traffic is the primary source of PM contamination during the inversion event sampled in December 2017. This conclusion is consistent with previous studies of Salt Lake City inversion events that relied on a combination of event‐specific aerial measurements and permanent station data to determine air quality for the entire Wasatch Front (Baasandorj et al., 2018). Other atmospheric chemistry studies focusing on Salt Lake City also indicate strong spatial heterogeneity with both gaseous (CO_2_, CH_4_, O_3_, and NO_2_) and PM_2.5_ concentrations increasing near busy roadways and intersections (Mitchell et al., 2018). Monaci et al. (2000) suggested that elevated levels of Ba and Zn on oak leaves nearest to motorways in Florence, Italy, indicate these metals can serve as a proxy for vehicular emissions. Our chemical results indicate elevated levels of Ba and (to a lesser extent) Zn at sites nearest to roads (Locations 1 and 3) consistent with their findings. Importantly, the lower SIRM values of the noninversion needles, as well as the lack of an appreciable relationship to nearby roads, suggest that evergreen needles have a sensitivity limit and therefore may not serve as reliable air quality samplers when PM concentrations fall beneath a specific but as yet undetermined threshold. In the week leading up to the 15 December 2017 poor air quality event, PM_2.5_ concentrations rose as high as 62.6 μg/m^3^ in Salt Lake City (Figure 2a). Conversely, PM_2.5_ concentrations never rose above ~10 μg/m^3^ during the 3.5 months leading up to the 14 June 2017 sampling of noninversion needles. Given these observations, it appears that at the sample volumes measured in this study (~10 cm^3^), needles are not reliable indicators of particulate matter pollution at or below ~10 μg/m^3^ levels. The results suggest that for PM concentrations at or above 62.6 μg/m^3^, rapid, low‐cost magnetic measurement of evergreen needles can be used to monitor air quality. At these sampling volumes, needles may be sensitive to PM concentrations below ~60 μg/m^3^, but additional study during less severe poor air quality events is needed to test this.

Previous studies that employed needles as passive monitors of air quality suggested that they provide a time‐averaged estimate of magnetic particulate matter contamination (Lehndorff et al., 2006; Lehndorff & Schwark, 2008; Urbat et al., 2004). Our study sought to evaluate the effectiveness of needles as recorders of short‐duration, acute air pollution events in urban environments. In the 6 months between sampling periods, the background PM_2.5_ concentration rarely exceeded ~20 μg/m^3^ until the inversion event prior to December sampling (Figure 2a). This suggests that the inversion event was the primary source for PM observed on needles collected on 15 December 2017. Additionally, several inversion events are observed in January and February 2017 but were not reflected in high SIRM or metal concentrations in the 14 June 2017 samples. This indicates that PM was removed from the needles during the intervening months prior to sampling, likely as a result of increased wind and precipitation in the spring (Figures 2b and 2c). This result suggests that if needles are indeed averaging pollution signals over the course of months or years as suggested by earlier studies (e.g., Urbat et al., 2004), then they may underestimate PM pollution in environments characterized by short‐term pollution events wherein PM accumulations are subsequently washed or stripped from the needles. For example, Chen et al. (2017) found that coniferous trees were less efficient at retaining PM during precipitation events than other species: conifers lost an average of 60% of deposited PM in comparison to 47% for broadleaf species. The authors also demonstrated a positive correlation between the amount of PM removed during precipitation events and the initial amount of PM present on the leaf. They suggested that because coniferous species are more efficient at scavenging PM from the atmosphere, they are also more susceptible to PM removal during precipitation. This appears to be consistent with our results, which indicate that although needles clearly scavenge PM during inversion events (December samples), they may well lose much of that PM over time due to strong precipitation and wind events (June samples). Magnetic analysis of evergreen needles over a longer period of time and with more frequent sampling intervals will better constrain the extent to which needles capture short duration poor air quality events, aggregate pollution from such events over time, and retain that PM through subsequent weather events (e.g., Hofman et al., 2017).

It is useful to compare SIRM result from this study to earlier studies of *P*. *nigra* needles in and around Cologne, Germany. Urbat et al. (2004) and Lehndorff et al. (2006) both measured the SIRM of needles collected from a variety of environments (busy roads, industrial regions, parks) during prolonged periods of no precipitation (at least 2 weeks). In the case of Urbat et al. (2004), the strongest SIRM values (~110 A m^−1^ kg^−1^) were measured in needles near an airport railway. Major roadways yielded a maximum SIRM of ~80 A m^−1^ kg^−1^, whereas the lowest SIRM value for the entire study was ~20 A m^−1^ kg^−1^. Noninversion SIRM results from our study agree well with background levels measured by Urbat et al. (2004) at ~20 A m^−1^ kg^−1^. However, our maximum SIRM value of 175 A m^−1^ kg^−1^ is more than twice as large as the most strongly magnetized roadside sample in Urbat et al. (2004). Given that both studies sample the same species of tree and the background SIRM values are similar between the studies, we conclude that this discrepancy is most likely the result of extreme air quality degradation associated with Salt Lake City inversion events which routinely surpass United States Environmental Protection Agency guidelines regarding short‐term particulate pollution levels (Utah Division of Air Quality 2017 Annual Report). Maximum SIRM values of PM‐laden needles measured by Lehndorff et al. (2006) are slightly lower (60–70 A m^−1^ kg^−1^) than the maximum values in our study (175 A m^−1^ kg^−1^). We suggest that these smaller maximum values are likely because Lehndorff et al. (2006) selected sites that were removed from obvious pollution point sources (such as busy motorways) in an effort to obtain more representative measures of air quality at each location. The minimum SIRM values reported in Lehndorff et al. (2006) (20–30 A m^−1^ kg^−1^) are similar to the minimum values we measured in our study (~20 A m^−1^ kg^−1^). We conclude that the similar background SIRM values between Cologne, Germany, and Salt Lake City, UT, confirm that *P*. *nigra* needles can be used as effective and efficient magnetic biomonitors of PM pollution.

### 4.2 Bulk Composition of Particulate Matter

Understanding the composition of PM pollution during inversion events is necessary for understanding its potential impact on health outcomes for Salt Lake Valley residents (Pope et al., 1999). Chemical analysis of particulate matter deposited onto the needles indicates that concentrations of several metals, many correlated with adverse health effects (e.g., Fe and Pb; Maher, 2019; Maher et al., 2008), increase during inversion events. As with the magnetic results, metal concentrations are especially enriched near roadways (Figure 5). The magnetic results clearly indicate increased concentrations of iron‐bearing particulate matter during inversion events. Chemical analysis shows that the concentration of a number of nonmagnetic elements correlates with iron concentrations. We find significant statistical correlations between SIRM, Na, Al, Cs, La, Ce, and Pb in samples collected during the December 2017 inversion event (Table S2). This suggests that magnetic analysis can be a proxy for the concentrations of toxic trace metals such as Cs and Pb, consistent with the conclusion of Maher et al. (2008) who conducted biomagnetic monitoring with deciduous tree leaves. Further development of needle magnetization as a robust proxy for other toxic elements could lead to a rapid and cost‐effective method for predicting elevated levels of toxic elements such as Pb throughout urban environments. This in turn could help assess how PM composition affects health outcomes in urban environments.

### 4.3 Composition and Grain Size of Magnetic Particles

The low‐temperature magnetic results help to constrain the composition of the magnetic material in particulate matter during inversion events. The dramatic decrease in remanence during cooling of a room temperature SIRM (Figure 4c) until ~110 K is diagnostic of magnetite undergoing a phase transition from cubic to monoclinic structure (i.e., the Verwey transition) (Verwey, 1939). The Verwey transition is typically observed at ~120 K, but partial oxidation of magnetite to maghemite shifts the transition to lower temperatures and can even obscure the Verwey transition entirely (Özdemir & Dunlop, 2010). We see further evidence of partial oxidation of magnetite in the pronounced “humped” shape of both cooling and warming curves above the Verwey transition in the low‐temperature RT‐SIRM experiments. This shape is the result of the combined low‐temperature behaviors of magnetite and maghemite and suggests the presence of particles cored by magnetite and surrounded by an oxidized maghemite shell (Bilardello, 2020; Özdemir & Dunlop, 2010). In this model, the oxidation process induces stress associated with coupling between the core and rim of the particle and causes the particle surface to fracture. This fracturing reduces the effective magnetic grain size of the maghemite on the surface. This conclusion is consistent with FC and ZFC results (Figure 4a) which show a rapid decrease in magnetization at ~20–25 K; this demagnetization behavior is consistent with unblocking of <20 nm superparamagnetic particles. We infer this behavior is due to the presence of small magnetic domains composed of maghemite on the surfaces of larger magnetite particles within the PM deposited onto the pine needles. Alternatively, or additionally, some of the particulate matter may consist of individual sub‐20 nm maghemite particles produced by fuel combustion, as documented by Maher et al. (2016).

That FC remanence is greater than ZFC remanence at low temperatures is another indication of small magnetic grain size. Smirnov (2009) conducted the same low temperature experiments on magnetite crystals ranging in size from 0.15 to 100 μm. His experiments documented an increase in the ratio between FC remanence and ZFC remanence at 20 K (termed R_LT_) with decreasing magnetic grain size (domain state). The R_LT_ value for our inversion sample is ~1.22 which corresponds to an average grain size of ~10 nm (Smirnov, 2009).

While these results indicate the presence of maghemite in the particulate matter, they do not uniquely distinguish between larger, “cracked” particles behaving magnetically as smaller grains or the presence of physically smaller (e.g., 5–15 nm) individual particles of maghemite and magnetite. Distinguishing between these morphologies is important given that health risk is in part a function of particle size (Könczöl et al., 2011). Direct imaging of individual particles with high magnification analytical transmission electron microscopy that can measure the oxidation state of iron (e.g., with electron energy loss spectroscopy; Gonet & Maher, 2019; Maher et al., 2016) will allow for particle size analysis and provide an independent test of the “maghemite shell” hypothesis described in the previous paragraph and directly observed in other studies (Ahmed & Maher, 2018). Quantifying differences (if any) between magnetic grainsize and physical grain size is extremely important in the context of human health risk. Although the results presented here do not provide a rigorous measure of grainsize (either physical or magnetic) in these samples, future work will target this outstanding question regarding the PM deposited onto the surface of evergreen needles.

### 4.4 Spatial Resolution of PM Sampling

Previous studies demonstrated the efficacy of evergreen needles in resolving spatial changes in pollution (Lehndorff & Schwark, 2009, 2010). For example, Lehndorff and Schwark (2009) collected needles from 71 trees situated ~5 km apart. They mapped pollution distributions in and around the Cologne Conurbation using three‐ring polycyclic aromatic hydrocarbon concentrations of PM deposited on tree needles. Our study was designed to monitor spatial changes in air quality on a much smaller scale (tens to hundreds of meters); the results described above indicate that evergreen needles are indeed capable of providing such spatial detail. The distance between Location 1 and Location 2 is approximately 50 m (Figure 1), yet the amount of PM contamination documented in both our magnetic and microscopy results suggest that the locations receive considerably different levels of particulate air pollution during poor air quality events. Salt Lake City (and many other high population regions) relies on a small number of air quality sensors to evaluate air quality and disseminate information to the public. Our results support the growing body of evidence that the true pattern of air quality in a given urban environment is complex and poorly represented by the small number of data points used by municipalities to make decisions regarding urban planning and human health (Caplin et al., 2019; Kelly et al., 2017; Mitchell et al., 2018; Wilson et al., 2005). Although this study focuses on only a single poor air quality event with a small number of sample sites (*n* = 4), the results confirm the need for high spatial resolution when quantifying air quality in polluted environments. Magnetic methods employed here appear capable of capturing the high degree of spatial variability in PM contamination during periods when air quality poses an increased risk to urban populations. The prevalence of evergreen trees throughout Salt Lake City and other communities in the region provides the potential for creating high spatial resolution maps of air quality during enhanced winter pollution events. Analytically and economically efficient biomagnetic monitoring using evergreen needles could improve our understanding of PM distribution on a city block scale or smaller.

### 4.5 Further Method Development

The results of the three methods employed to measure metal concentrations (acid leaching, sonication, and total digestion) can be explained in a few different ways. We observe that for the majority of metals measured, concentrations are highest in digested needles (Figures 6a–6d). We suggest that this indicates one of two possibilities: (1) neither acid leaching nor sonication in water is effective at removing all metal particulate matter from the surfaces of the needles, or (2) a substantial fraction of the measured metals is contained beneath the surface of the needle and is only extracted after total needle digestion and not by surface cleaning. The first option is important as we develop and refine the use of evergreen needles as particulate air pollution samplers. Lehndorff and Schwark (2008, 2010) analyzed trace elements of needles via total digestion from samples collected in Cologne, Germany. We can compare our results directly to these studies by summing our digest and acid leach results from Location 1; this is because our digested samples were first measured via acid leaching to quantify surface concentrations (see Section 2.3 for details). The results from this study and Lehndorff and Schwark (2008) are similar for most measurements, including Fe concentration (~200 mg/kg) and Sb (~0.5 mg/kg), V (~0.3 mg/kg), Cd (0.02–0.05 mg/kg), Ti (2–5 mg/kg), and Mo (0.4–0.9 mg/kg). Our results indicate that ~70–95% of these metals are measured in needle digestion after leaching (Figure 6d). Although our results do not definitively indicate where these particles accumulate (e.g., within wax layers or in stomata), they do support previous studies that suggest PM may be bound tightly to the needle through particle encapsulation via wax degradation (Terzaghi et al., 2013) or incorporation into stomatal cavities (Urbat et al., 2004). Additional work studying the timescales of these processes relative to removal processes would help clarify their significance for the preservation of PM accumulated during short‐term poor air quality events experienced during atmospheric inversion.

If metals deposited onto tree needles (and other plant tissues, e.g., bark) are incorporated into those plant tissues, then this raises important considerations for human health in urban environments (Mosbaek et al., 1989; Uzu et al., 2010; Xiong et al., 2017). If trees remove hazardous particulate matter from polluted environments by incorporating that material into their tissues (especially needles and leaves), then planting additional trees in regions subject to frequent and acute poor air quality events can help remove inhalable PM from the air (Rindy et al., 2019; Wang et al., 2019). For example, Chen et al. (2017) studied the efficiency of PM_2.5_ capture across a range of tree species and found that coniferous trees accumulated the most PM (>20 μg/cm^2^), whereas the smoothest broadleaf species retained the least. Urban forestation may be especially important for marginalized populations who are often disproportionately affected by poor air quality (Mullen et al., 2020; O'Neill et al., 2003). While our results confirm that evergreen trees show promise in this regard, it is important to critically evaluate the variety of additional factors involved in planned urban forestation (Escobedo et al., 2011; Grote et al., 2016). Conversely, consumption of produce grown in polluted urban environments in personal or community gardens may result in increased consumption of toxic metals and additional health concerns associated with atmospheric pollutant contamination (Uzu et al., 2014). The results of the study presented here support the conclusion that heavy metal contamination in the tissues of urban produce is a function of both proximity to roadways (Antisari et al., 2015) and soil contamination (Cooper et al., 2020). Strategic urban forestation can mitigate these effects.

Given the importance of particulate matter grain size to health outcomes, a more detailed understanding of the particle size distribution of PM on evergreen needles is critical. High‐resolution analytical transmission electron microscopy provides visual determination of particle size in the critical size range (tens of nanometers) and will also reveal particle oxidation state and provide additional chemical information. There are several low temperature magnetic experiments which are sensitive to magnetic grain size and have been used to quantify grainsize distributions. These methods could allow for more robust statistical treatment of particle size than would be possible with TEM analysis alone. For example, thermal fluctuation tomography (Jackson et al., 2006) has quantified magnetic grainsize distributions in natural samples containing <20 nm‐sized superparamagnetic material. The grainsize distributions of Jackson et al. (2006) were determined for Tiva Canyon Tuff and are comparable to independent measures of grainsize for the same rocks (Schlinger et al., 1988, 1991; Till et al., 2011). Although these results suggest that thermal fluctuation tomography may be extremely useful in the context of ultra‐fine magnetic PM distributions in air pollution studies, it has yet to be applied to samples that show a high degree of oxidation and the potential for substantial differences between physical and magnetic grainsize as discussed earlier. This additional complexity can be evaluated and addressed with concomitant high‐resolution TEM analyses.

## 5 Conclusions

Our results are consistent with previous studies that show magnetic measurements applied to evergreen needles serve as proxies for particulate matter pollution in urban environments. The apparent high degree of spatial resolution afforded by biomagnetic monitoring using pine needles is particularly important for developing spatial maps of PM during poor air quality events. The use of evergreen needles allows for year‐round monitoring of air quality, which is especially important for intermontane population centers where winter‐time air quality can be especially poor due to atmospheric temperature inversions. When coupled with deployable particle size analyzers, these methods can quickly and rigorously quantify the spatial heterogeneity of PM pollution. Chemical analyses indicate the enrichment of multiple metal contaminates during inversion in Salt Lake City. These chemical analyses also show a correlation between iron‐bearing (magnetic) particles and nonmagnetic but toxic elements such as lead and cesium. Therefore, the magnetization of evergreen needles might also be a proxy for other toxin loadings in the environment. Our results indicate that needles are capable of capturing relatively short duration (3–4 day) periods of severely degraded air quality and also suggest that long‐term PM retention may underestimate the magnitude of these events. Continuous biomagnetic monitoring of evergreen needles over the course of several months or years and through multiple precipitation and poor air quality events will better constrain the effectiveness and temporal resolution of our analysis.

## Conflict of Interest

The authors declare no conflicts of interest relevant to this study.

## Supporting information

Supporting Information S1

## Data Availability

Data presented in this paper are available for download from the figshare data repository (doi: 10.6084/m9.figshare.12790106).
