# Supplementary material for: Evergreen Needle Magnetization as a Proxy for Particulate Matter Pollution in Urban Environments
Source: Geohealth. 2020 Sep 10;4(9):e2020GH000286. doi: 10.1029/2020GH000286 (PMC7507380; doi:10.1029/2020GH000286)
Supplement: Supplementary file 1 — Supporting Information S1 [file GH2-4-e2020GH000286-s001.pdf]

Supporting Information

Evergreen needle magnetization as a proxy for particulate matter pollution in urban environments

Grant Rea-Downing[1], Brendon J. Quirk[1,2†], Courtney L. Wagner[1], Peter C. Lippert[1,3]*

[1]*Department of Geology and Geophysics, University of Utah, Salt Lake City, Utah, USA*

[2†]*Now at the Department of Geosciences, University of Massachusetts, Amherst, Massachusetts, USA*

[3]*Global Change and Sustainability Center, University of Utah, Salt Lake City, Utah, USA*

* Corresponding authors: grant.readowning@utah.edu, pete.lippert@utah.edu

Supporting information table of contents:

Number of figures: Figure S1

Number of tables: Tables S1, S2, S3, S4, S5

Number of pages: S1-S11

List of Figures

Figure S1: Metal concentrations measured on needles from Location 1 (top) and Location 4 (bottom) collected during inversion and non-inversion.

List of Tables

Table S1: Metal concentrations in ppm for acid leached needles from Locations 1-4 both during inversion and non-inversion. Calculated enrichment factors based upon measured concentrations are shown to the right. BLoD indicates concentrations below instrument detection limits. Detection limits are listed to the right of each element name. # BLoD indicates the number of measurements for which concentrations were below detection limits for each aliquot.

Table S2: Table showing the Pearson correlation coefficient for each elemental and/or SIRM pair. Bold values indicate statistically significant results with $p \leq 0.05$.

Table S3: Metal concentrations in ppm for sonicated (left) and microwave digested (right) needles from Locations 1 and 4. Abbreviations follow those in Table S1.

Table S4: Evergreen needle magnetization data for non-inversion and inversion samples.

Table S5: Low temperature magnetic data collected from needles sampled from Location 1 both non-inversion and during inversion. Abbreviations as described in SI 1: Low temperature magnetic methods.

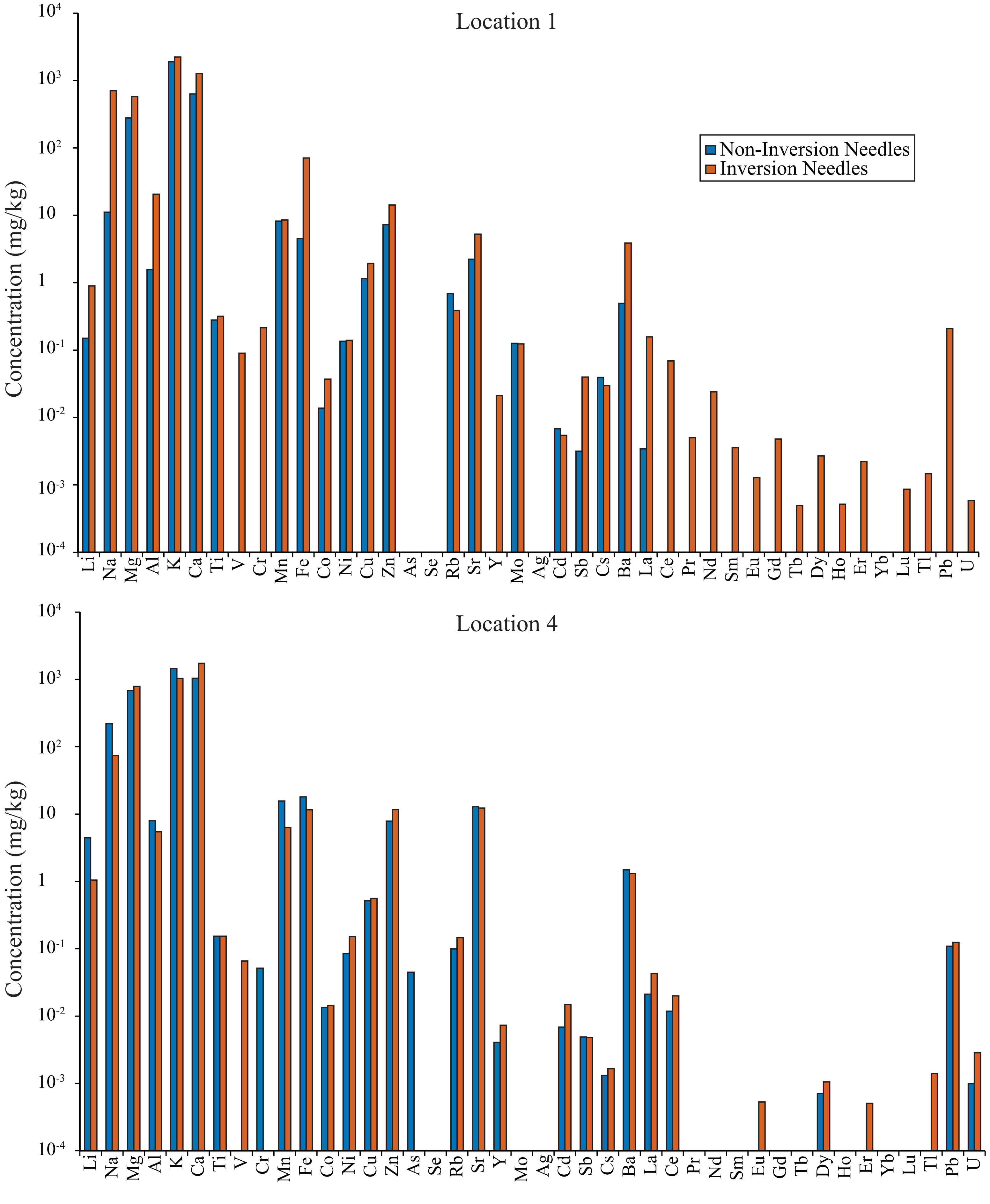
Location 1
Location 4
Non-Inversion Needles
Inversion Needles
Concentration (mg/kg)
10^4
10^3
10^2
10
1
10^-1
10^-2
10^-3
10^-4
Li Na Mg Al K Ca Ti V Cr Mn Fe Co Ni Cu Zn As Se Rb Sr Y Mo Ag Cd Sb Cs Ba La Ce Pr Nd Sm Eu Gd Tb Dy Ho Er Yb Lu Tl Pb U

Table S1

		Leachate (ppm)								Enrichment Factor			
Element	LoD	L1 Inversion	L1 Non-Inversion	L2 Inversion	L2 Non-Inversion	L3 Inversion	L3 Non-Inversion	L4 Inversion	L4 Non-Inversion	L1	L2	L3	L4
Li	0.06	0.90	0.15	0.61	1.01	2.86	1.28	1.04	4.44	6.0	0.6	2.2	0.2
Na	7	708	11	96	48	175	14	75	220	63.4	2.0	12.9	0.3
Mg	0.3	581.3	277.9	618.1	762.1	902.6	487.4	786.1	680.2	2.1	0.8	1.9	1.2
Al	0.112	20.6	1.6	8.3	9.0	14.3	6.9	5.4	7.9	13.2	0.9	2.1	0.7
K	9	2241	1904	1509	2189	1406	2337	1029	1459	1.2	0.7	0.6	0.7
Ca	11	1266	632	1198	1851	2075	960	1729	1039	2.0	0.6	2.2	1.7
Ti	0.03	0.32	0.28	0.18	0.22	0.14	0.11	0.15	0.15	1.14	0.81	1.27	1.00
V	0.04	0.09	BLoD	BLoD	BLoD	BLoD	BLoD	0.07	BLoD	N/A	N/A	N/A	N/A
Cr	0.02	0.21	BLoD	BLoD	BLoD	BLoD	BLoD	BLoD	0.05	N/A	N/A	N/A	N/A
Mn	0.0709	8.5336	8.2160	9.4933	15.2114	13.7216	8.8304	6.2830	15.5967	1.0387	0.6241	1.5539	0.4028
Fe	0.2	70.9	4.5	18.2	18.2	21.7	12.0	11.5	18.0	15.6	1.0	1.8	0.6
Co	0.0006	0.0371	0.0138	0.0192	0.0214	0.0171	0.0113	0.0144	0.0134	2.6932	0.8997	1.5098	1.0700
Ni	0.02	0.14	0.14	0.12	0.09	0.08	0.06	0.15	0.09	1.04	1.30	1.26	1.78
Cu	0.008	1.933	1.146	0.716	0.804	0.456	0.409	0.559	0.516	1.686	0.890	1.113	1.084
Zn	0.01	14.26	7.23	11.62	13.99	16.62	11.02	11.64	7.86	1.97	0.83	1.51	1.48
As	0.02	BLoD	BLoD	BLoD	BLoD	BLoD	BLoD	BLoD	0.04	N/A	N/A	N/A	N/A
Se	0.02	BLoD	BLoD	BLoD	BLoD	BLoD	BLoD	BLoD	BLoD	N/A	N/A	N/A	N/A
Rb	0.001	0.386	0.687	0.212	0.219	0.122	0.147	0.146	0.100	0.561	0.969	0.829	1.461
Sr	0.004	5.258	2.229	6.235	10.685	17.728	8.149	12.279	12.838	2.359	0.584	2.175	0.956
Y	0.0007	0.0212	BLoD	0.0081	0.0043	0.0064	0.0034	0.0073	0.0041	N/A	1.8934	1.8854	1.7893
Mo	0.04	0.12	0.13	BLoD	BLoD	BLoD	BLoD	BLoD	BLoD	N/A	N/A	N/A	N/A
Ag	0.002	BLoD	BLoD	BLoD	BLoD	BLoD	BLoD	BLoD	BLoD	N/A	N/A	N/A	N/A
Cd	0.002	0.005	0.007	0.005	0.013	0.009	0.007	0.015	0.007	0.807	0.392	1.171	2.165
Sb	0.002	0.040	0.003	0.010	0.006	0.008	BLoD	0.005	0.005	12.536	1.637	N/A	0.982
Cs	0.0006	0.0298	0.0392	0.0046	0.0026	0.0071	0.0021	0.0017	0.0013	0.7592	1.7481	3.3707	1.2640
Ba	0.006	3.867	0.493	1.949	2.670	4.437	1.902	1.308	1.485	7.842	0.730	2.333	0.881
La	0.002	0.157	0.003	0.070	0.017	0.104	0.017	0.043	0.021	46.109	4.086	6.010	2.026
Ce	0.0055	0.0692	BLoD	0.0260	0.0107	0.0409	0.0099	0.0199	0.0118	N/A	2.4321	4.1181	1.6941
Pr	0.0013	0.0050	BLoD	BLoD	BLoD	BLoD	BLoD	BLoD	BLoD	N/A	N/A	N/A	N/A
Nd	0.0052	0.0240	BLoD	BLoD	BLoD	BLoD	BLoD	BLoD	BLoD	N/A	N/A	N/A	N/A
Sm	0.0015	0.0035	BLoD	BLoD	BLoD	BLoD	BLoD	BLoD	BLoD	N/A	N/A	N/A	N/A
Eu	0.0002	0.0013	BLoD	0.0010	BLoD	0.0008	BLoD	0.0005	BLoD	N/A	N/A	N/A	N/A
Gd	0.0008	0.0048	BLoD	BLoD	BLoD	BLoD	BLoD	BLoD	BLoD	N/A	N/A	N/A	N/A
Tb	0.0001	0.0005	BLoD	BLoD	BLoD	0.0003	BLoD	BLoD	BLoD	N/A	N/A	N/A	N/A
Dy	0.0005	0.0027	BLoD	0.0016	BLoD	BLoD	BLoD	0.0011	0.0007	N/A	N/A	N/A	N/A
Ho	0.0001	0.0005	BLoD	BLoD	BLoD	BLoD	BLoD	BLoD	BLoD	N/A	N/A	N/A	N/A
Er	0.0003	0.0022	BLoD	0.0011	BLoD	0.0006	BLoD	0.0005	BLoD	N/A	N/A	N/A	N/A
Yb	0.0006	BLoD	BLoD	BLoD	BLoD	BLoD	BLoD	BLoD	BLoD	N/A	N/A	N/A	N/A
Lu	0.0001	0.0009	BLoD	BLoD	BLoD	BLoD	BLoD	BLoD	BLoD	N/A	N/A	N/A	N/A
Tl	0.0007	0.0015	BLoD	BLoD	0.0024	0.0166	0.0062	0.0014	0.0019	N/A	N/A	N/A	N/A
Pb	0.02	0.21	BLoD	0.12	0.11	0.16	0.09	0.12	0.11	N/A	1.07	1.84	1.14
U	0.0002	0.0006	BLoD	0.0008	0.0019	0.0022	0.0012	0.0028	0.0010	N/A	0.3994	1.8576	2.8759
# BLoD		4	21	15	17	14	18	13	14				

Table S2

Inversion Samples

	SIRM	Li	Na	Mg	Al	K	Ca	Ti	Mn	Fe	Co	Ni	Cu	Zn	Rb	Sr	Y	Cd	Sb	Cs	Ba	La	Ce	Eu	Er	Pb
Li	-0.05																									
Na	**0.99**	-0.16																								
Mg	-0.48	0.86	-0.54																							
Al	**0.96**	0.21	0.91	-0.27																						
K	0.95	-0.22	0.93	-0.67	0.90																					
Ca	-0.33	0.89	-0.40	**0.99**	-0.13	-0.56																				
Ti	0.92	-0.45	**0.95**	-0.77	0.77	0.93	-0.65																			
Mn	0.07	0.82	-0.07	0.50	0.36	0.08	0.50	-0.27																		
Fe	**0.99**	-0.20	**1.00**	-0.59	0.90	**0.96**	-0.45	**0.97**	-0.06																	
Co	**0.96**	-0.31	**0.98**	-0.69	0.86	**0.97**	-0.57	**0.99**	-0.11	**0.99**																
Ni	0.10	-0.83	0.24	-0.58	-0.19	0.08	-0.56	0.43	**-0.98**	0.23	0.28															
Cu	0.92	-0.43	**0.96**	-0.74	0.78	0.92	-0.62	**1.00**	-0.28	**0.97**	**0.99**	0.44														
Zn	0.45	0.86	0.34	0.49	0.68	0.31	0.59	0.06	0.82	0.31	0.21	-0.74	0.08													
Rb	0.87	-0.53	0.91	-0.84	0.71	0.92	-0.74	**0.99**	-0.30	0.93	**0.97**	0.46	**0.99**	-0.03												
Sr	-0.42	0.89	-0.50	**1.00**	-0.21	-0.61	**0.99**	-0.74	0.55	-0.54	-0.65	-0.63	-0.71	0.56	-0.81											
Y	0.94	-0.38	**0.97**	-0.70	0.79	0.92	-0.57	**0.99**	-0.27	**0.98**	**0.98**	0.43	**1.00**	0.11	**0.97**	-0.67										
Cd	-0.58	0.20	-0.53	0.64	-0.62	-0.80	0.62	-0.60	-0.35	-0.59	-0.65	0.24	-0.56	-0.19	-0.64	0.59	-0.52									
Sb	**0.97**	-0.30	**0.99**	-0.67	0.86	**0.96**	-0.54	**0.99**	-0.14	**0.99**	**1.00**	0.31	**0.99**	0.21	**0.97**	-0.63	**0.99**	-0.60								
Cs	**0.99**	-0.17	**1.00**	-0.57	0.91	**0.95**	-0.43	**0.96**	-0.04	**1.00**	**0.99**	0.21	**0.96**	0.34	0.92	-0.52	**0.97**	-0.58	**0.99**							
Ba	0.67	0.67	0.56	0.21	0.85	0.58	0.31	0.33	0.76	0.55	0.47	-0.65	0.34	**0.95**	0.25	0.28	0.36	-0.45	0.47	0.57						
La	**0.97**	0.14	0.92	-0.35	**1.00**	0.93	-0.22	0.81	0.32	0.93	0.89	-0.15	0.82	0.62	0.76	-0.29	0.83	-0.66	0.89	0.94	0.82					
Ce	**0.99**	0.07	**0.96**	-0.38	**0.99**	0.93	-0.24	0.86	0.20	**0.96**	0.93	-0.02	0.87	0.56	0.81	-0.32	0.88	-0.59	0.93	**0.97**	0.76	**0.99**				
Eu	0.84	-0.27	0.81	-0.73	0.80	**0.97**	-0.65	0.85	0.15	0.85	0.89	0.00	0.83	0.23	0.87	-0.68	0.81	-0.92	0.86	0.85	0.51	0.85	0.82			
Er	0.90	-0.45	0.92	-0.81	0.78	**0.97**	-0.71	**0.99**	-0.17	0.95	**0.98**	0.33	**0.97**	0.07	**0.99**	-0.77	**0.96**	-0.72	**0.97**	0.94	0.36	0.83	0.85	0.93		
Pb	**0.98**	0.15	**0.95**	-0.28	**0.98**	0.88	-0.13	0.82	0.20	0.94	0.89	-0.03	0.83	0.61	0.75	-0.22	0.85	-0.49	0.90	0.95	0.78	**0.97**	**0.99**	0.74	0.79	
U	-0.62	0.48	-0.61	0.85	-0.56	-0.84	0.83	-0.75	-0.04	-0.67	-0.75	-0.08	-0.71	0.05	-0.80	0.81	-0.67	0.95	-0.71	-0.66	-0.25	-0.63	-0.59	-0.94	-0.85	-0.48

Non-Inversion Samples

	SIRM	Li	Na	Mg	Al	K	Ca	Ti	Mn	Fe	Co	Ni	Cu	Zn	Rb	Sr	Cd	Cs	Ba
Li	0.53																		
Na	0.46	**0.97**																	
Mg	**0.96**	0.54	0.53																
Al	**1.00**	0.52	0.43	**0.95**															
K	0.05	-0.74	-0.85	-0.10	0.08														
Ca	0.80	0.03	0.06	0.86	0.79	0.29													
Ti	-0.59	-0.56	-0.33	-0.38	-0.61	-0.09	-0.05												
Mn	0.80	0.66	0.73	0.92	0.77	-0.44	0.71	-0.15											
Fe	**0.97**	0.65	0.62	**0.99**	**0.97**	-0.16	0.77	-0.50	0.91										
Co	0.43	-0.22	-0.08	0.60	0.42	0.18	0.87	0.44	0.59	0.46									
Ni	-0.76	-0.35	-0.14	-0.55	-0.78	-0.37	-0.38	0.92	-0.24	-0.62	0.12								
Cu	-0.69	-0.62	-0.41	-0.49	-0.70	-0.05	-0.16	**0.99**	-0.28	-0.61	0.34	0.93							
Zn	0.67	-0.27	-0.33	0.62	0.68	0.70	0.88	-0.18	0.33	0.54	0.69	-0.55	-0.23						
Rb	-0.91	-0.67	-0.53	-0.79	-0.92	0.01	-0.49	0.86	-0.61	-0.87	-0.03	0.90	0.92	-0.44					
Sr	0.94	0.78	0.73	0.93	0.93	-0.27	0.61	-0.62	0.87	**0.98**	0.26	-0.66	-0.72	0.38	-0.92				
Cd	0.54	-0.25	-0.17	0.65	0.54	0.37	0.94	0.27	0.54	0.52	**0.97**	-0.10	0.17	0.85	-0.17	0.32			
Cs	**-0.96**	-0.58	-0.44	-0.84	**-0.96**	-0.09	-0.61	0.81	-0.63	-0.90	-0.16	0.90	0.87	-0.57	**0.99**	-0.91	-0.31		
Ba	0.90	0.10	0.03	0.84	0.91	0.45	0.91	-0.43	0.58	0.80	0.60	-0.72	-0.50	0.93	-0.73	0.70	0.76	-0.83	
La	0.94	0.74	0.62	0.85	0.94	-0.12	0.53	-0.80	0.71	0.92	0.10	-0.83	-0.87	0.41	**-0.99**	**0.97**	0.21	**-0.98**	0.72

Table S3

Element	LoD	Sonicate (ppm)				LoD	Digest (ppm)			
		L1 Inversion	L1 Non-Inversion	L4 Inversion	L4 Non-Inversion		L1 Inversion	L1 Non-Inversion	L4 Inversion	L4 Non-Inversion
Li	0.00039	0.15	BLoD	0.11	0.13	0.004	2.63	0.22	2.74	10.14
Na	0.0005	102	7	16	11	0.3	2399	24	189	279
Mg	0.00001	70.4	6.1	63.1	46.0	0.002	1918.3	1228.4	2983.4	2071.8
Al	0.000005	77.8	0.9	17.4	28.3	0.005	114.9	5.3	14.2	28.3
K	0.00745	145	209	90	57	4	6394	8822	4809	5050
Ca	0.013	432	47	861	557	0.4	7824	2857	6801	7389
Ti	0.00002	3.28	0.12	0.58	0.98	0.001	5.53	2.08	2.75	2.21
V	0.00002	0.24	0.01	0.05	0.06	0.001	0.24	0.01	0.04	0.05
Cr	0.00001	0.42	0.01	0.07	0.07	0.001	0.77	0.04	0.48	0.59
Mn	0.00002	1.8660	0.5059	0.9056	1.1290	0.003	24.5573	59.5185	29.0023	50.1967
Fe	0.0001	124.0	1.3	17.2	25.5	0.002	141.0	33.7	35.9	38.3
Co	0.000002	0.0545	0.0008	0.0120	0.0128	0.00002	0.1110	0.0754	0.0626	0.0488
Ni	0.000004	0.27	0.03	0.47	0.08	0.0003	0.46	0.60	0.25	0.23
Cu	0.00003	2.703	0.220	1.152	0.326	0.001	5.474	7.257	4.960	2.529
Zn	0.00002	11.33	0.73	3.18	1.94	0.006	39.39	40.74	45.85	28.22
As	0.00003	0.10	0.04	0.04	0.05	0.001	0.13	0.16	0.07	0.09
Se	0.00003	0.01	BLoD	0.01	0.01	0.002	0.04	BLoD	0.02	0.01
Rb	0.00001	0.152	0.099	0.050	0.058	0.02	1.164	3.603	0.810	0.427
Sr	0.000001	1.028	0.264	6.383	4.430	0.0001	25.592	5.655	36.852	42.031
Y	3E-07	0.0471	0.0008	0.0121	0.0176	0.00001	0.0676	0.0030	0.0100	0.0140
Mo	0.00001	0.11	0.01	0.02	0.01	0.001	0.79	2.99	0.68	0.27
Ag	2.8E-06	0.005	0.011	0.002	0.002	0.0001	0.020	0.005	0.013	0.030
Cd	3.6E-06	0.003	BLoD	0.004	0.003	0.0001	0.022	0.025	0.065	0.029
Sb	2.1E-06	0.354	0.003	0.019	0.024	0.0002	0.558	0.006	0.023	0.033
Cs	1.2E-06	0.0145	0.0004	0.0037	0.0058	0.005	0.0422	0.0041	0.0037	0.0055
Ba	2.5E-06	6.585	0.104	0.743	0.787	0.0002	8.632	1.221	3.593	3.653
La	2.1E-06	0.402	0.002	0.105	0.091	0.00001	0.422	0.007	0.082	0.090
Ce	1.8E-06	0.1808	0.0031	0.0450	0.0624	0.00002	0.2920	0.0478	1.3204	0.0768
Pr	4E-07	0.0157	0.0002	0.0041	0.0056	0.000005	0.0201	0.0006	0.0030	0.0054
Nd	2.1E-06	0.0701	BLoD	0.0160	0.0223	0.00003	0.0895	0.0029	0.0116	0.0186
Sm	2.6E-06	0.0109	BLoD	0.0030	0.0039	0.00003	0.0254	0.0048	0.2135	0.0074
Eu	6E-07	0.0039	BLoD	0.0007	0.0010	0.00001	0.0054	0.0005	0.0015	0.0016
Gd	1.5E-06	0.0099	BLoD	0.0025	0.0035	0.00002	0.0136	0.0007	0.0149	0.0032
Tb	2E-07	0.0014	BLoD	0.0003	0.0005	0.000005	0.0017	BLoD	0.0006	0.0004
Dy	1.4E-06	0.0069	BLoD	0.0020	0.0027	0.00002	0.0092	BLoD	0.0019	0.0020
Ho	3E-07	0.0013	BLoD	0.0004	0.0006	0.000005	0.0018	BLoD	0.0004	0.0004
Er	1.1E-06	0.0037	BLoD	0.0009	0.0014	0.00001	0.0049	BLoD	0.0009	0.0012
Yb	1.7E-06	0.0035	BLoD	0.0008	0.0013	0.00002	0.0042	BLoD	0.0008	BLoD
Lu	3E-07	0.0005	BLoD	0.0001	0.0002	0.000004	0.0006	BLoD	0.0001	BLoD
Tl	8E-07	0.0014	BLoD	0.0004	0.0004	0.00002	0.0066	0.0004	0.0022	0.0053
Pb	4.8E-06	0.37	0.02	0.19	0.14	0.0002	0.59	0.10	0.16	0.18
U	5E-07	0.0057	0.0003	0.0101	0.0051	0.0007	0.0120	0.0011	0.0155	0.0103
# BLoD		0	14	0	0		0	7	0	2

Table S4

Step (mT)	Non-Inversion Needle Magnetization (A/m)			
	Location 1	Location 2	Location 3	Location 4
0 (NRM)	1.17E-04	2.36E-05	5.39E-05	5.40E-06
20	1.15E-03	2.27E-03	1.31E-03	1.84E-03
50	2.88E-03	1.05E-02	5.61E-03	8.25E-03
100	5.11E-03	2.00E-02	1.14E-02	1.66E-02
300	6.37E-03	3.19E-02	1.15E-02	2.18E-02
650	6.71E-03	3.38E-02	1.43E-02	2.24E-02
1000 (SIRM)	6.89E-03	2.59E-02	1.45E-02	1.95E-02
	Inversion Needle Magnetization (A/m)			
Step (mT)	Location 1	Location 2	Location 3	Location 4
0 (NRM)	1.97E-05	1.92E-04	6.18E-04	5.46E-05
20	1.57E-02	6.59E-03	5.83E-03	2.64E-03
50	6.98E-02	2.06E-02	3.31E-02	1.05E-02
100	1.46E-01	3.47E-02	5.74E-02	1.91E-02
300	2.04E-01	4.20E-02	7.51E-02	2.38E-02
650	2.02E-01	4.15E-02	6.58E-02	2.48E-02
1000 (SIRM)	2.02E-01	3.72E-02	7.26E-02	2.46E-02

Table S5

Location 1 Non-Inversion FC, remanence		Location 1 Non-Inversion ZFC, remanence		Location 1 Non-Inversion RT remanence cooling		Location 1 Non-Inversion RT remanence warming	
T [K]	M [Am2/kg]	T [K]	M [Am2/kg]	T [K]	M [Am2/kg]	T [K]	M [Am2/kg]
19.99	0.000030322	20.00	0.000026195	299.99	0.000014000	19.99	0.000011630
25.51	0.000027732	25.57	0.000023996	294.53	0.000014088	25.57	0.000011310
30.53	0.000025957	30.59	0.000022450	289.30	0.000014051	30.66	0.000010826
35.63	0.000025773	35.54	0.000021882	284.27	0.000014044	35.59	0.000011401
40.53	0.000022694	40.48	0.000020750	279.13	0.000013981	40.62	0.000011947
45.48	0.000021960	45.52	0.000019902	274.20	0.000013721	45.49	0.000012466
50.51	0.000022035	50.62	0.000020558	269.16	0.000014002	50.55	0.000012296
55.53	0.000020356	55.64	0.000019237	264.23	0.000013986	55.57	0.000012343
60.59	0.000019575	61.68	0.000018550	259.24	0.000013801	60.58	0.000012288
65.62	0.000020345	65.65	0.000020683	254.29	0.000013743	65.60	0.000012863
70.44	0.000019465	70.52	0.000017578	249.26	0.000014224	70.51	0.000012565
75.45	0.000018783	75.51	0.000017754	244.30	0.000014034	75.51	0.000012584
80.59	0.000018393	80.58	0.000017902	239.38	0.000013791	80.58	0.000012035
85.49	0.000018567	85.60	0.000017169	234.25	0.000014235	85.51	0.000012227
90.59	0.000017035	90.55	0.000017062	229.34	0.000014007	90.69	0.000011765
95.66	0.000017111	95.64	0.000017152	224.38	0.000014505	95.61	0.000012683
100.58	0.000016500	100.63	0.000016094	219.36	0.000013694	100.53	0.000011651
105.45	0.000016812	105.57	0.000016249	214.26	0.000013982	105.48	0.000011635
110.63	0.000016013	110.58	0.000016339	209.29	0.000013489	110.53	0.000011558
115.55	0.000016947	115.53	0.000015636	204.33	0.000013489	115.59	0.000011847
120.56	0.000015877	120.53	0.000015608	199.37	0.000013815	120.60	0.000012395
125.62	0.000015079	125.61	0.000015238	194.32	0.000013914	125.60	0.000011443
130.52	0.000015401	130.45	0.000015051	189.30	0.000013864	130.52	0.000012196
135.51	0.000015400	135.58	0.000015085	184.40	0.000013600	135.57	0.000012699
140.59	0.000015016	140.63	0.000013807	179.27	0.000013473	142.81	0.000012333
145.61	0.000014332	145.52	0.000014456	174.44	0.000013689	145.49	0.000011941
150.54	0.000014455	150.52	0.000014954	169.34	0.000013860	150.60	0.000011698
155.54	0.000013777	155.56	0.000014970	164.34	0.000013965	155.61	0.000012268
160.48	0.000014223	160.50	0.000013759	159.30	0.000013523	160.51	0.000012744
165.50	0.000015179	165.52	0.000013717	154.36	0.000013479	165.57	0.000012249
170.71	0.000014022	170.61	0.000014563	149.38	0.000014030	170.53	0.000011599
175.58	0.000014062	175.52	0.000013386	144.29	0.000012423	175.49	0.000012526
180.50	0.000014815	180.60	0.000013350	139.32	0.000013702	180.61	0.000011387
185.61	0.000013089	187.76	0.000013742	134.31	0.000014555	185.57	0.000012039
190.50	0.000011857	190.69	0.000011923	129.31	0.000013936	190.53	0.000011795
195.47	0.000012992	195.58	0.000013311	124.35	0.000014028	195.54	0.000012308
200.62	0.000012465	200.60	0.000013310	119.33	0.000012701	200.52	0.000011803

205.53	0.000011284	205.59	0.000013811	114.39	0.000014187	205.56	0.000012390
210.52	0.000012637	210.62	0.000013418	109.37	0.000012686	210.66	0.000012312
215.62	0.000012425	215.55	0.000012969	104.32	0.000011631	216.67	0.000012072
220.49	0.000012184	220.60	0.000012755	99.20	0.000012172	220.53	0.000011846
225.54	0.000012801	225.60	0.000012286	94.35	0.000013800	225.53	0.000011995
230.72	0.000012321	230.66	0.000011737	89.19	0.000012993	230.66	0.000011579
235.62	0.000012283	235.56	0.000012725	84.27	0.000011321	235.61	0.000012063
240.58	0.000011241	240.56	0.000012620	79.35	0.000011827	240.58	0.000011561
245.59	0.000011952	245.58	0.000012491	74.24	0.000012720	245.70	0.000011746
250.51	0.000011388	250.48	0.000012325	69.24	0.000012795	250.57	0.000011792
255.58	0.000011977	255.56	0.000012178	64.30	0.000013144	255.56	0.000011690
260.60	0.000011692	260.60	0.000011307	59.30	0.000012485	260.54	0.000011638
265.55	0.000011014	265.60	0.000011388	54.15	0.000009948	265.57	0.000011796
270.51	0.000011229	270.62	0.000011843	49.34	0.000013021	270.54	0.000011776
275.67	0.000011235	275.66	0.000011716	44.23	0.000012639	275.57	0.000011302
280.45	0.000010806	280.57	0.000011528	39.24	0.000013113	280.59	0.000011692
285.55	0.000010835	286.52	0.000011294	34.26	0.000012237	285.46	0.000012032
290.58	0.000010743	292.77	0.000011113	29.23	0.000012225	290.64	0.000011514
295.48	0.000010504	295.51	0.000010990	24.21	0.000014820	295.51	0.000011587
300.03	0.000010219	300.02	0.000010713	20.00	0.000012122	300.02	0.000011530

Location 1 Inversion FC, remanence		Location 1 Inversion ZFC, remanence		Location 1 Inversion RT remanence cooling		Location 1 Inversion RT remanence warming	
T [K]	M [Am2/kg]	T [K]	M [Am2/kg]	T [K]	M [Am2/kg]	T [K]	M [Am2/kg]
20.00	0.001077405	20.01	0.000885755	300.01	0.000226984	20.00	0.000205098
25.55	0.001003215	25.57	0.000818102	294.66	0.000227022	25.55	0.000206369
30.53	0.000892486	30.62	0.00073081	289.27	0.000227461	30.58	0.000206514
36.60	0.000797469	35.64	0.000665718	284.26	0.000228557	35.62	0.000205397
40.63	0.000747673	40.55	0.000616639	279.05	0.000228331	40.61	0.000206376
45.55	0.000689524	45.61	0.000574017	274.23	0.000229638	45.53	0.000205978
50.64	0.000633631	50.67	0.000537033	269.36	0.000229452	50.69	0.000204526
55.64	0.000601744	55.53	0.000513565	264.33	0.000230277	55.56	0.000206354
60.49	0.000575938	60.60	0.000490495	259.26	0.00023068	60.52	0.000206399
65.61	0.000546216	65.57	0.000471006	254.40	0.000230963	65.60	0.000205858
70.51	0.000520995	70.53	0.000452997	249.61	0.000230788	70.53	0.000206142
75.57	0.000496928	75.52	0.000432739	244.56	0.000231415	75.52	0.000206119
80.57	0.000472125	80.57	0.000414424	239.48	0.000230704	80.57	0.000206149
85.55	0.000455322	85.58	0.00039981	234.39	0.000231673	85.52	0.000205755
90.44	0.000437247	90.58	0.000385221	229.50	0.000230991	90.56	0.000206317
95.63	0.000416551	95.58	0.000372077	224.48	0.000232012	95.70	0.000206078
100.55	0.00040671	100.50	0.000361975	219.44	0.000230306	100.52	0.000206613
105.52	0.000391323	105.49	0.000350676	214.45	0.000230821	105.53	0.000206131

110.66	0.000378263	110.59	0.000338482	209.36	0.000230391	110.60	0.000206494
115.53	0.000366284	115.54	0.000330155	204.32	0.000230483	115.57	0.000206734
120.57	0.000354604	120.68	0.000319721	199.47	0.000230648	122.56	0.000206609
125.64	0.000328403	125.59	0.000311032	194.51	0.000230453	125.59	0.000206308
130.57	0.00033223	130.54	0.000302921	189.35	0.000230833	130.51	0.000206887
135.52	0.000323003	135.52	0.000296832	184.38	0.000230417	135.57	0.000207452
140.65	0.000304686	140.66	0.00027461	179.49	0.00022984	140.65	0.00020779
145.52	0.000310113	145.65	0.000287047	174.39	0.00022928	145.60	0.000208174
150.56	0.000303379	150.48	0.000281352	169.42	0.000229667	150.52	0.000208024
155.62	0.000273129	155.63	0.000255856	164.49	0.000229473	155.64	0.000207515
160.50	0.000291951	160.55	0.000272002	159.36	0.000228474	160.47	0.000207157
165.57	0.000286153	165.58	0.000267052	154.49	0.00022652	165.67	0.000207491
170.70	0.000269573	170.58	0.000245263	149.52	0.000224828	170.71	0.000206844
175.52	0.000276129	175.58	0.000259783	144.51	0.000224499	175.62	0.000206401
180.48	0.000270456	180.56	0.000254595	139.36	0.00022353	180.61	0.000207068
185.71	0.000256298	185.68	0.000236576	134.45	0.000221644	185.68	0.000206384
190.59	0.000262292	190.56	0.000248635	129.41	0.000220365	190.52	0.000205932
195.59	0.000255746	195.53	0.000244561	124.42	0.00021778	195.56	0.000207176
200.68	0.000245013	200.66	0.000234055	119.49	0.000215647	200.70	0.000206053
205.54	0.00024819	205.52	0.000237796	114.52	0.000214038	205.51	0.000205679
210.52	0.000243545	210.50	0.000234455	109.40	0.000210889	210.57	0.000206089
215.72	0.000237292	215.62	0.000228974	104.44	0.000210585	215.64	0.000204691
220.46	0.000236775	220.52	0.000228777	99.39	0.000210061	220.52	0.000205086
226.61	0.000231873	225.49	0.000224157	94.34	0.000209699	225.53	0.000204921
230.64	0.000228323	230.61	0.000220388	89.43	0.000209426	230.56	0.000203844
235.60	0.000226444	235.61	0.000219624	84.30	0.000209484	235.52	0.000203571
240.58	0.000223406	240.52	0.000216672	79.29	0.000208486	240.56	0.000204128
245.59	0.000219027	245.64	0.000213441	74.29	0.000208182	245.55	0.000203108
250.56	0.000216052	250.54	0.000211033	69.22	0.000207774	250.57	0.000202252
255.63	0.000212785	255.57	0.000208988	64.27	0.000206899	255.57	0.000202317
260.57	0.000209787	260.58	0.000205218	59.28	0.000207343	260.61	0.000200517
265.52	0.000207008	265.53	0.000203064	54.25	0.000206886	265.53	0.000200613
270.54	0.000204896	270.62	0.000201082	49.35	0.00020656	270.50	0.000199958
275.67	0.000201764	275.64	0.000198179	44.43	0.000206419	275.59	0.000200236
280.57	0.000197966	280.46	0.000195451	39.29	0.000206574	280.53	0.000198775
285.49	0.000194982	285.61	0.000193506	34.33	0.000206456	285.60	0.0001978
290.65	0.000190952	290.69	0.000190296	29.31	0.000206651	290.58	0.000197294
295.60	0.000188956	297.80	0.000186679	24.19	0.000206526	295.60	0.000196778
300.02	0.000182231	300.03	0.000181265	19.99	0.000205042	300.03	0.000193923
